# Evolutionary significance of antiparasite, antipredator and learning phenotypes of avian nest defence

**DOI:** 10.1038/s41598-018-28275-3

**Published:** 2018-07-12

**Authors:** Daniela Campobello, Spencer G. Sealy

**Affiliations:** 1 Department of Biological Sciences, University of Manitoba, Winnipeg, MB Canada; 2 Present Address: Section of Animal Biology, Department STEBICEF, University of Palermo, Palermo, Italy

## Abstract

Avian nest defence, which is expected to serve both antiparasite and antipredator functions, may benefit or be detrimental to birds, although selective forces that potentially operate on nest defence have not been quantified as a whole. Together with fitness values, we analysed two traits of nest defence, intensity and plasticity, in two distantly related passerine species, yellow warbler (*Setophaga petechia*) in North America and reed warbler (*Acrocephalus scirpaceus*) in Europe, both favourite host species for brood parasites. Breeders that escaped parasitism were the most vocal among reed warblers, whereas there was no specific defence phenotype that predicted prevention of parasitism in yellow warblers. Breeders that escaped nest predation were, in both species, those with the most distractive response at the first exposure to a nest-threatening event, such as the experimental predation or parasitism simulated at the nest. However, increasing defence intensity benefited yellow warblers but was detrimental to reed warblers, because intense defence responses attracted predators. Adaptiveness of nest defence was revealed by nest defence phenotypes when examined in concert with the seasonal fitness (i.e. measures of reproductive success). Results revealed selective forces favoured yellow warblers with strong defence phenotypes. Opposite forces were instead revealed among reed warblers whose favoured phenotypes were strong, yet less flexible, defenders.

## Introduction

Nest defence is one of the frontline defences that allows birds to preserve their fitness against threatening attacks of brood parasites and nest predators^1–4^. Thus, antiparasite and antipredator are two different functions expected from an effective nest defence. Preventing an event of parasitism may, however, require responses that differ from protecting the nest from a nest predation attack. These requirements can be mutually exclusive so they trade off against each other^4^. For example, physical attacks may be effective to prevent parasitism but should be avoided when confronting a nest predator, which is often a predator also of nesting adults.

Studies on the adaptiveness of nest defence as an antiparasite or antipredator strategy have revealed contrasting results^5^. Nest defence appears to effectively deter parasitism, whereas in other systems it may attract predators^6,7^. Other scenarios have not been addressed, such as whether nest defence directed toward predators may attract parasites. In all cases defence responses have been correlated to fitness proxies such as either parasitism or predation rates^7–9^. However, parasitism and predation have rarely been analysed together in the same system^10,11^; therefore, only partial assessments are available of the efficacy of nest defence as a complex and multifunctional behaviour. Distraction displays, alarm calls and direct attacks are only some of the behavioural responses included within mobbing defensive strategies adopted by breeders to protect their nests from parasites and predators^1,12^. Combinations of these and other responses together with their intensity level may depict different defence profiles or phenotypes^13^.

Despite many studies showing that nest defence is advantageous for reducing predation rate^2,4,14–17^, we do not know whether, and to what extent, different defence phenotypes are under selection. Selection would occur if variation in defence phenotypes entails variation in fitness, with nest defence variability, therefore driving important evolutionary implications as a result of triggering selective processes at the population level^18^. To reveal potential fitness variation correlated to defence phenotypes, it is necessary to analyse measures of fitness as a cumulative result of all functional performances of defence (i.e. both antiparasite and antipredator performances), so that we can assess quality and quantity of potential selective forces in progress^19,20^.

Here, our main goal was to quantify the potential selection acting on avian defence phenotypes. We approached it by adopting a phenotypic selection analysis that allowed us to examine multilevel selection of correlated traits and quantify strength and direction of selection with simple selection metrics^21,22^. As largely recommended for testing the efficacy of a multifunction behaviour, we adopted a more holistic approach that simultaneously examined multiple traits in independent phyletic groups^23^.

We analysed two phenotypic traits of nest defence, intensity and plasticity, shown by breeders during experimental trials. The intensity of a defence response was quantified as the number of defence responses (e.g. alarm calls or attacks) to nest enemies at the first threat of exposure. We here refer to defence plasticity as the change in defence intensity that some species exhibited after exposure to an experimental nest-threatening event, such as parasitism or predation^24–26^. Defence change recorded in the field has proven to be an excellent model to quantify the learning ability of avian species in wild populations and therefore to assess their phenotypic plasticity^2,12,27,28^. Modulation of nest defence intensity according to environmental pressures may occur if individuals can acquire information, personally or by observing conspecifics, about nest threats in their surroundings and change the expression of defence accordingly^29,30^. Such a change has been described as phenotypic plasticity that is modulated via learning abilities (*sensu*^31^) by parasitism and/or predation pressure^4^. Both intensity and plasticity of nest defence are continuous traits which, as such, exhibit a continuum of defence phenotypes. Here, for brevity, we define weak to strong defenders the phenotype range for defence intensity and learner to non-learner defenders the phenotype range for defence plasticity.

Using experimental and comparative approaches, we analysed defence traits in two distantly related passerine birds that defend nests against both avian brood parasites and predators^25,32,33^. We examined nest defence of yellow warblers (*Setophaga petechia*), parasitized by the brown-headed cowbird (*Molothrus ater*) in North America, and reed warblers (*Acrocephalus scirpaceus*), parasitized by the common cuckoo (*Cuculus canorus*) in Europe. Both species discriminate between parasites and other threatening or non-threatening species by responding with stimulus-specific behaviours^32,33^. Furthermore, both species show learning abilities applied to defence expressions by increasing the intensity of antiparasite defence after experiencing simulated events of parasitism or predation^12,27,28^. Behavioural changes are mediated by learning in both yellow^27^ and reed^12,28^ warblers.

To reveal the selective forces on nest defence, we included two more specific objectives. First, the effectiveness of nest defence: we tested whether intensity and plasticity defence phenotypes involved significant fitness differences in terms of nest survival time, and, second, nest defence selection: we quantified the strength and direction of selective forces acting on intensity and plasticity of defence phenotypes. If nest defence was an effective strategy against both parasitism and predation, we expected the highest nest survival among the strongest defenders in the population. We also expected that individuals that rapidly learn to increase defence intensity would enjoy higher nest survival than non-learners. With respect to different fitness benefits among defence intensity and plasticity phenotypes, we expected high and positive selection gradients for both defence traits, indicating that enemies at the nest have exerted selection for strong and plastic defence strategies. We discuss these results within the context that nest defence involves social interactions within and among host species and, as such, triggers coevolutionary responses in predators and parasites.

## Results

### Yellow warblers

Nesting yellow warblers experienced cumulative frequencies of parasitism of 13% (out of 338 nests) and predation of 65% (out of 425 nests), respectively; thus, a cumulative frequency of survival to parasitism of 0.87 (±SE, ±0.02, Kaplan-Meier Analysis) and to egg predation of 0.35 (±0.06).

#### Effectiveness of nest defence phenotypes

**Figure 1:**
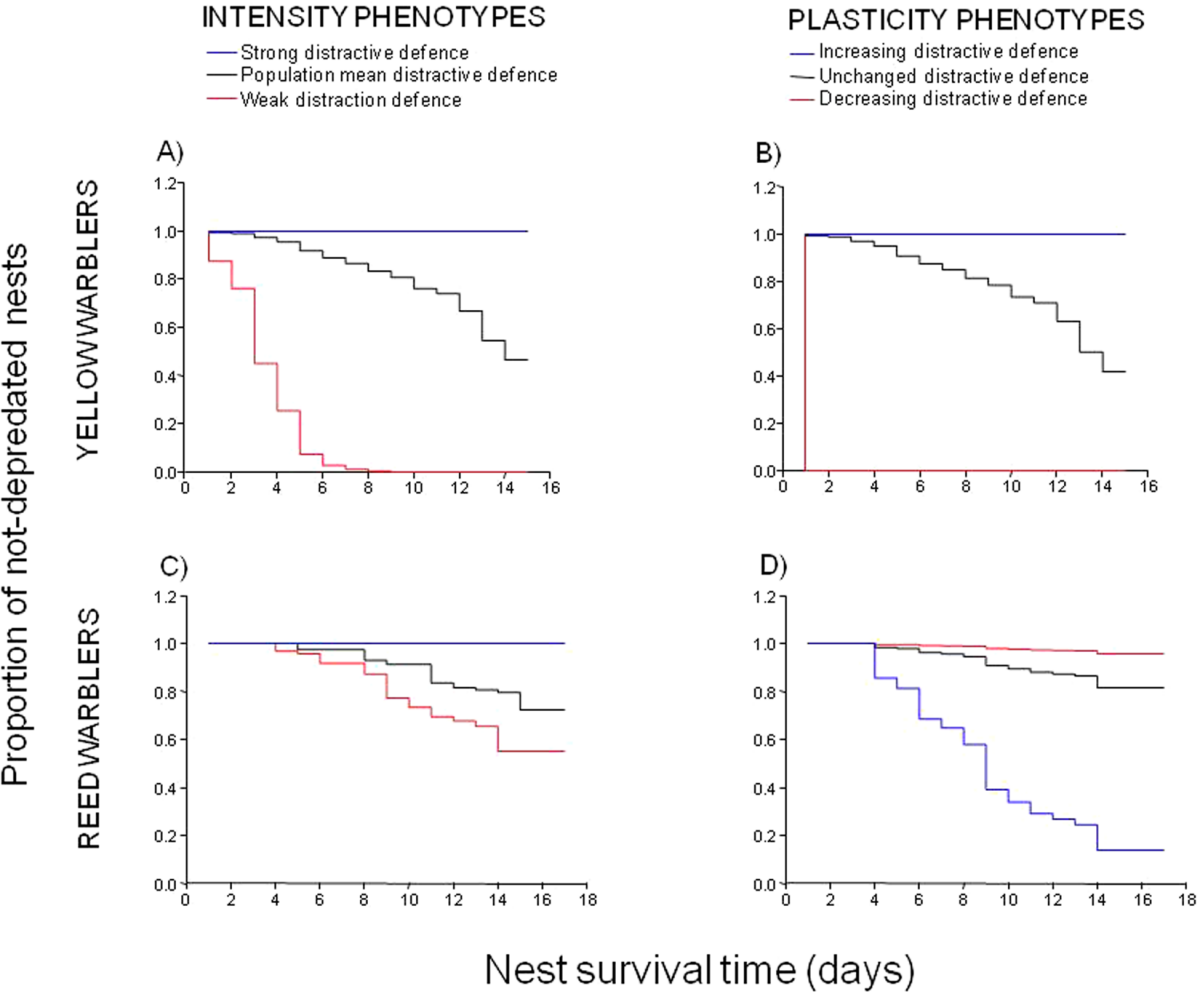
Proportion of nests that remained unpredated until hatching shown by nest survival curves per species and defence phenotypes. Cox models indicated a distractive defence (e.g. number of perch changes) as an effective antipredator strategy in both species first exposed to nest threat. Survival curves (**A**,**C**) show breeders with strongest defence (i.e. blue lines) were those with the highest hatching occurrence (i.e. number of nests remained not depredated until hatching). Plasticity (i.e. change of defence intensity after the first nest threat exposure) of distractive defence was as well a significant predictor of an antipredator strategy but with opposite effects in the two species. Yellow warblers that increased their defence intensity most benefited from high nest survival rates to predator attacks (**B**), contrary to the same plasticity phenotypes of reed warblers (**D**) that instead suffered the highest level of nest predation. In yellow warblers intense and plastic phenotypes of a protective defence (e.g. nest-protection behaviour) and plastic phenotypes of aggressive defence (e.g. strikes to nest enemy models, Supplementary Fig. 1) resulted in effective antipredator strategies with similar nest survival curves shown for the distractive defence (**A**,**B**).

No intensity or plasticity phenotype significantly deterred parasitism, thus no defence phenotype predicted survival of nests to parasitism (all Cox Models, χ_2_ = 0.002–3.070, df = 4, P > 0.05, N = 164). By contrast, according to one model, three mutually exclusive defence strategies (Supplementary Table 1, Supplementary Figure 1) – distractive, protective and aggressive defences – resulted in significant predictors of nest survival to predation (Cox Model, χ_2_ = 54.43, df = 24, P = 0.0004, N = 199). Yellow warbler breeders responding with many distraction displays during initial model presentations (i.e. strong defenders as intensity phenotype) or those increasing them during later exposures (i.e. learner defenders as plasticity phenotype) significantly escaped egg predation (Fig. 1A,B).Yellow warblers that reacted either with perch changes and *chip* calls or by sitting tightly in their nests (i.e. nest-protection behaviour) with high intensities during the first model presentation or successive exposures incurred significantly less egg predation than both weaker and non-learning conspecific defenders. Nest survival curves showed benefits accrued by strong defenders versus both weak defenders and those using an average defence intensity (Fig. 1A). Also, nests of learners survived longer than non-learners (Fig. 1B).

#### Selection of nest defence phenotypes

**Figure 2:**
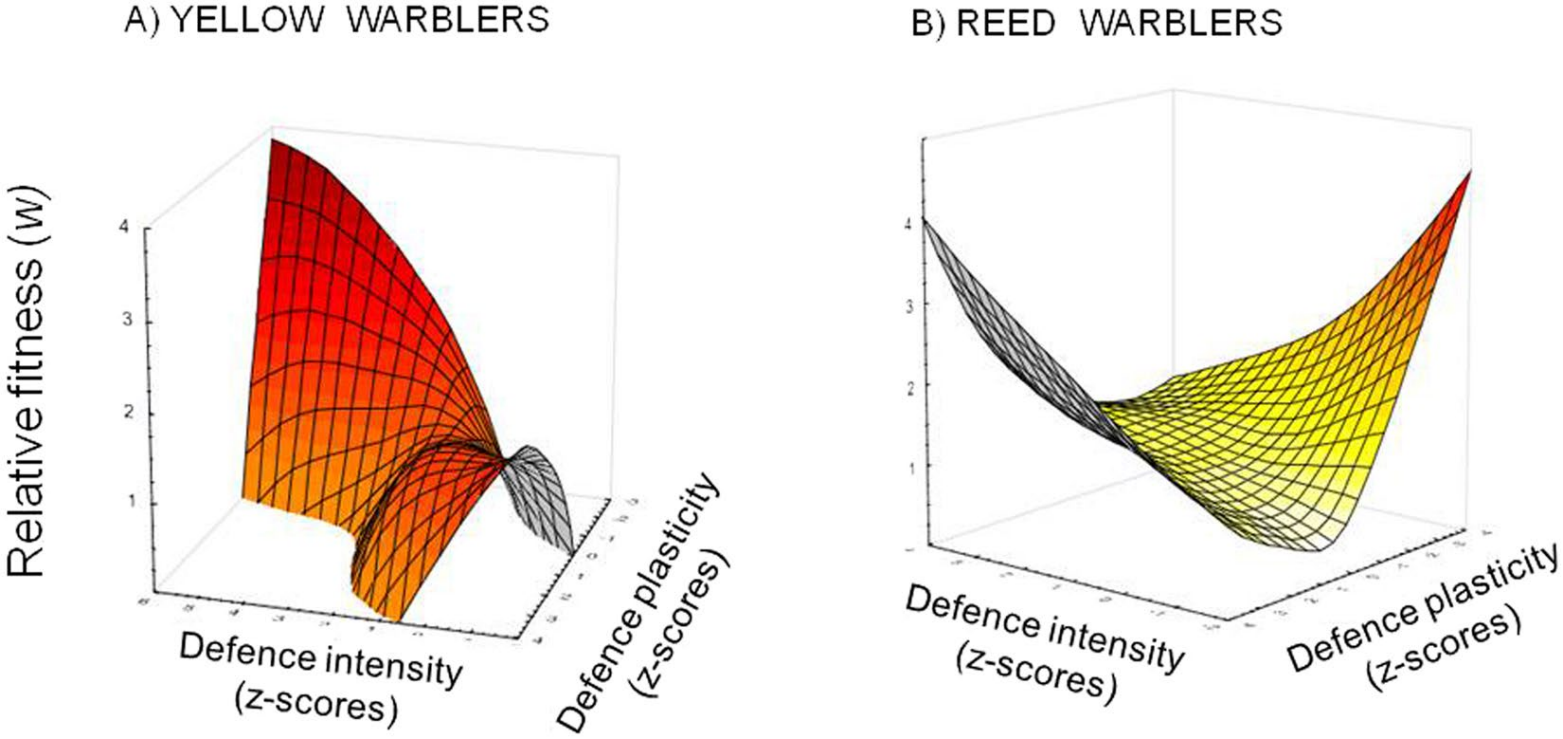
Thin plate splines representing fitness surfaces of (**A**) yellow warblers and (**B**) reed warblers. Relative fitness *w* is a function of the interaction between intensity and plasticity of defence phenotypes measured as standardized values (z-scores) of defence responses. Strength of selection acting on intensity and plasticity phenotypes is quantified in Table 1.

Multilevel selection analyses showed that fitness values (i.e. the number of hatchlings) were not significantly associated with either intensity or plasticity of defence activity responses (all P values > 0.05). By examining effect sizes, while nonlinear selection presented γ selection gradients with low values for both defence traits, linear selection showed values of β selection gradients that suggest a moderate/high selection pressure toward a more intense defence response (Table 1A). Graphical representation revealed a saddle-shaped fitness surface (Fig. 2A), with a bivariate curvature typical of correlation selection^32^, and thus maximum fitness values were achieved by individuals exhibiting a combination of defence phenotypes that implied either a strong, yet little flexible, defence in the first and last threat exposures, or a weak response at first, but increasingly strong subsequently. Both the intensity β value (Table 1) and the fitness surface (Fig. 2A) indicated that the absolute best strategy was the first one, that is selection favoured strong yet inflexible defence intensity, rather than phenotypic plasticity in learning a more intense defence.

**Table 1:** Partial regression coefficients, indicating the effect size of linear (β) and nonlinear(γ) selection forces acting on intensity and plasticity defence phenotypes of (A) yellow warblers and (B) reed warblers.

	LINEAR	NONLINEAR
(A) YELLOW WARBLERS	F_2,54_ = 1.40 R² = 0.049	F_2,54_ = 0.69 R² = 0.025
	β ±SE	γ ±SE
Intensity	0.234 ±0.14	0.095 ±0.14
Plasticity	0.151 ±0.14	0.112 ±0.14
(B) REED WARBLERS	F_2,84_ = 2.30 R² = 0.052	F_2,84_ = 0.28 R² = 0.007
	β ±SE	γ±SE
Intensity	0.107 ±0.11	0.015 ±0.11
Plasticity	−0.166 ±0.11	0.076 ±0.11

Partial β selection gradients estimate the effect of each phenotype on fitness, whereas γ selection gradients on quadratic terms show the forces acting on phenotypic variance (i.e. γ < 0 indicates a decreasing phenotypic variance implying stabilizing selection, γ > 0 indicates an increasing phenotypic variance implying disruptive selection).As strong selective pressures are usually described as those with 0.15–0.30 cumulative selection gradients (reviewed in^16^), here we revealed important directional selection forces favouring stronger and more plastic phenotypes of yellow warbler defence (A), whereas almost no directional selection on nest defence of reed warblers (B). Among these, the resulting negligible selection strength is, however, the result of opposite forces operating on either phenotype. In other words, the opposite signs of β selection gradients show that, while defence intensity might be favoured, its plasticity during successive threat encounters is not. All γ nonlinear selection gradients showed small effect sizes indicating no presence of either stabilizing or disruptive selection acting on defence phenotypes of both species.

### Reed warblers

Nesting reed warblers experienced a cumulative parasitism rate of 24% (out of 169 nests) and predation rate of 29% (out of 187 nests); that is, their nests showed a cumulative frequency of survival to parasitism of 0.76 (±0.09, Kaplan-Meier Analysis) and to egg predation of 0.71 (±0.05).

#### Effectiveness of nest defence phenotypes

**Figure 3:**
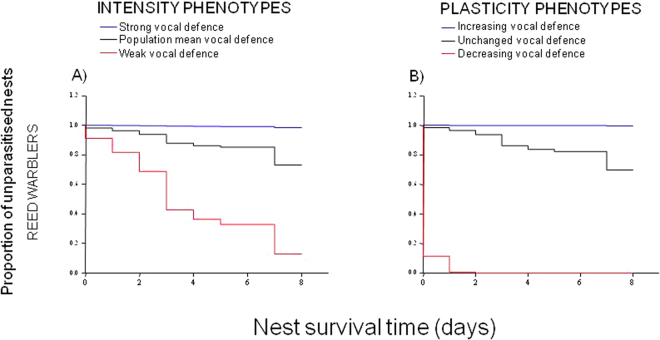
Survival curves of the proportion of nests that escaped parasitism in reed warblers. Cox models indicated a vocal defence (e.g. number of alarm *rasp* calls) as an effective antiparasite strategy not only among the most vocal breeders (**A**, blue lines) but also in those that most increased their vocal response after the first threat exposure (**B**). In yellow warblers, there was no equivalent defence response that significantly predicted nest survival to parasite attacks, indicating no effective antiparasite strategy adopted by breeders.

Contrary to yellow warblers, the intensity and plasticity defence phenotypes were associated with breeders that significantly escaped parasitism and, specifically, high frequency of *rasp* alarm calls predicted which nests escaped parasitism. The strongest intensity (Ws = 6.26, P = 0.012, N = 117, Fig. 3A) and greatest plasticity (Ws = 6.55, P = 0.010), in terms of increasing number of *rasp* calls, Fig. 3B), were recorded at unparasitised nests, indicating that individuals that were highly vocal when first exposed, or that increased the number of *rasp* calls when later presented with a brood parasite, more often escaped parasitism than less vocal individuals.

Intensity and plasticity phenotypes of a distractive defence (i.e. number of perch changes) were significant predictors of which nests escaped egg predation, although with contrasting effects. As for yellow warblers, strong defence phenotypes were mostly associated with non-depredated nests, thus the stronger the distractive defence the longer the nest survived to egg predation (Ws = 11.22, P = 0.00008, N = 121, Fig. 1C). Contrary to yellow warblers, however, plastic phenotypes revealed that increased defence intensity during successive presentations had a slight probability that their nest was the target of nest predators (Ws = 3.87, P = 0.049, Fig. 1D). There was an indication that this might have been the result of predators attracted by other birds recorded during first presentation as their presence was negatively associated with nest survival to egg predation (Ws = 5.89, P = 0.015).

#### Selection of nest defence phenotypes

Multilevel selection analyses showed that fitness values (i.e. the number of hatchlings) were not significantly associated with either intensity or plasticity of defence activity responses (all P values > 0.05). Also, by examining effect sizes, selection gradients suggested a moderate/low selection pressure; however, β values showed opposite selection directions for intensity (positive) and plasticity (negative) of defence, indicating that strong responses in the first place provided a fitness advantage but not if the intensity increased when exposed again (Table 1). Graphical representation showed a convex fitness surface (Fig. 2B), which indicates two potential best combinations of defence phenotypes. However, given the small and opposite effect sizes shown by the selection gradients (Table 1), selection forces as depicted by this fitness surface (Fig. 2B) were weak or negligible.

## Discussion

Our results revealed complex selection forces acting on avian nest defence in its dual roles of antiparasite and antipredator functions. Despite many convergent similarities, we showed, within two species belonging to different parasitic/predator systems, defence phenotypes that have been differentially selected so that we could detect from neutral to opposite selection strengths and directions (Table 2 for a summary).

**Table 2:** Qualitative summary of results divided per focal species, defence phenotypes and selection parameters.

Species	Nest defence function	Intensity phenotypes	Plasticity phenotypes	nest survival effect^a^ (predictor)	linear selection^b^ (direction and strength)
		nest survival effect^a^ (predictor)	linear selection^b^ (direction and strength)		
Yellow warbler	Antiparasite	0	+strong	0	+mild
	Antipredator	+distraction		+distraction	
		+protection		+protection	
				+aggression	
Reed warbler	Antiparasite	+alarm *rasp* calls	+weak	+alarm *rasp* calls	−mild
	Antipredator	+distraction		−distraction	

^a^Nest survival effect: + and − indicated whether the behavioural predictor aside was associated with a longer or shorter time of nest survival to parasite or predator attacks, respectively, whereas 0 indicates no effect of any phenotype on nest survival times.

^b^Linear selection direction referred to the sign of the β selection gradients (Table 1), thus + selection forces favouring stronger and faster learners, whereas − favouring the opposite phenotypes, thus weaker and slower learners. We arbitrarily categorised selection strength according to a previous literature review of β selection gradients (reviewed in^18^) as strong > 0.20, mild = 0.15–0.20, weak < 0.15.

Our first result regarding intensity phenotypes was unexpected as suggested by previous investigations^6,33^: all intensity phenotypes, weak to strong, provided no antiparasite benefits in yellow warblers, contrary to strong defence phenotypes of reed warblers, where the most vocal breeders were parasitized the least. Strong defence phenotypes provided antipredator advantages to both species sharing the most distractive defence as the one associated with the fewest depredated nests.

Results for plastic phenotypes, from learners to non-learners, depicted a more diverse scenario than suggested previously^12,27^. When examining the antiparasite function, results mirrored those of intensity phenotypes. Once again, all plastic phenotypes seemed to provide no antiparasite benefits in yellow warblers, contrary to reed warbler learners where breeders learning to increase alarm calls were those with the fewest parasitized nests. As for their antipredator function, defence phenotypes sorted in the least and most depredated nests in yellow and reed warblers, respectively. Specifically, yellow warblers with increased distraction, protection and aggression displays avoided nest predation, whereas reed warblers with increased distraction displays met with increased predation at their nests. Future investigations are warranted to determine whether the mechanisms underlying changes in defence (i.e. learning *sensu*^31^) are related to age or experience.

Results of selective forces acting on phenotypes of nest defence, examined cumulatively^21,34^ as antiparasite and antipredator behaviour, revealed stronger selection in progress in yellow than in reed warblers. Yellow warblers that reacted strongly in the first place were more favoured than those increasing their defence successively, although a mild positive selection force also prevailed on the learner phenotype. Despite association with longer nest survival (i.e. nest neither parasitised nor depredated), selection forces toward stronger reed warblers were weak. This is probably because of the opposite effects revealed by increasing defence intensity, learner phenotype was mildly selected against in reed warblers, as results revealed.

In summary, the only result that supported our hypothesis was positive selection toward strong yellow warbler defenders. The other results, mostly unexpected, beg two questions. In yellow warblers, why is there no defence phenotype able to avoid brood parasitism? In both species, why increasing defence intensity is not strongly selected despite the evolution and maintenance of learning for modulating nest defence responses?

Across thirty years, in our yellow warbler population, parasitism rate has been recorded with an annual average of 18%^35^, a selection pressure that should result in the evolution of antiparasite defences^36^ if compared with other parasitized populations^37^. Each breeding attempt is, however, mainly challenged by nest predators. Generalist predators, including red-winged blackbird (*Agelaius phoeniceus*), common grackle (*Quiscalus quiscula*), and American red squirrel (*Tamiasciurus hudsonicus*) have annually depredated an average of 47% of warbler nests, with peaks of 63% and 71% when including nestling predation^35^. This impact of nest predation together with the extreme fluctuations recorded in the annual parasitism rates may explain the absence of phenotypes to contrast parasitism in favour of evolving a rich repertoire of antipredator defences, which in fact we revealed as three alternative strategies (i.e. distractive, protective and aggressive defence).

Despite its occurrence in the form of individual learning in yellow warblers^27^ and social learning in reed warblers^12,28^, defence plasticity was not strongly favoured, with selection only mildly favouring yellow warbler learners and even hindering learning by reed warblers. This last result is explained in reed warblers as we revealed opposite effects of their nest defence as antiparasite and antipredator roles. Increased predation recorded with an increased defence intensity may, in turn, be supported by the effect revealed by the main antiparasite call (i.e. *rasp* call), thus attracting neighbours^4,28^ among which there may also be predators^1,6^, although we are still unaware of which species of predator were attracted. Taken together, the contrasting effects of a stronger nest defence negated any selective benefit of increased defensive intensities.

Yellow warblers also possess a rich repertoire elicited by parasites and predators and, therefore, it was puzzling we did not detect a similar increase in predation with increasing defence intensity recorded in reed warblers. This may be supported by the absence of neighbours recruited by alarm calls and the less likelihood of attracting predators, but we lack such records. Yellow warblers, however, nest at very high densities at Delta Marsh, so the acoustic properties of their main alarm calls may easily be eavesdropped by nesting conspecifics^24^. One explanation supporting the absence of conspecific recruitment is the discarding of social cues^35^, which in fact is what yellow warblers do by not changing the intensity of their defence even following repeated exposure to social cues^38^. Contrary to reed warblers, which, as a population trait, rely on public information, yellow warblers use personal cues only to adjust their defence intensity. This has been explained by the extensive annual fluctuation of nest threatening events across thirty years (reviewed in^35^, as great environmental variation is commonly invoked as the main cause of rendering public information obsolete and therefore unreliable^39–42^). Although the absence of a neighbour, and possibly a predator, not eliciting recruitment among conspecifics is consistent with the learning mode adopted by yellow warblers, it is a question that has not been investigated. Other factors such as a short coevolutionary history with the parasite and less virulence of cowbird parasitism may prevent or retard the evolution of antiparasitic defences in yellow warblers.

In providing the first quantification of selective forces acting on an animal’s learnable behaviour, our study revealed the advantages of behavioural learning and that its differential selection may have important implications for recent findings on animal conformism^43^ and extended social phenotypes^44^. In the first case, Aplin *et al*.^43^ showed that individuals are predisposed to conform to the most common response used by conspecifics. This may explain animals using social rather than individual learning independent of environmental fluctuations. In the second case, as recently reported for interspecific groups of nesting avian species, selective forces also operate in extended group phenotypes^44^. Learning, here resulting in suboptimal or even maladaptive effects^38^, is a social trait, and, as such, the analysis of its role in a more comprehensive coevolutionary scenario cannot be complete without being examined as part of an extended group phenotype such as multi-species defence responses. Our results also provided support for selection forces acting on nest defence that might shape coevolutionary trajectories^45^ within predator/prey and parasite/host systems. Our approach offers interesting avenues to better define the role of learning of avian species exposed to both parasitism and predation triggering coevolutionary changes^46^.

## Methods

### Study species and field protocol

We quantified nest-defence responses of yellow warblers at Delta Marsh (Portage la Prairie, MB, Canada) from May to June in 2002 and 2003 and of reed warblers at the Valli di Mortizzuolo (hereafter Tomina, Modena, Italy) from April to June in 2004 and 2005. Within our broader investigation on both species^27,28^, we searched for warbler nests and tagged them with numbered tape for reference on visits every 1–3 days, weather permitting, until clutch completion. During nest inspections we recorded clutch size, parasitism and egg predation, and number of hatchlings. Parasitism was obvious during nest inspection because of the different size and colour of the parasite egg compared with those of the host species,

The yellow warbler is among the most important host species of the brood-parasitic brown-headed cowbird. Parasitized yellow warblers seldom fledge more than one chick versus 3–4 from unparasitised nests. Because of their smaller size, yellow warbler chicks in fact generally starve to death as the result of the monopolization of food by their larger foster cowbird “sibling“^47^. The reed warbler is among the common cuckoo’s preferred hosts in Europe, including in Italy^4,48^. Cuckoo parasitism proves extremely costly to hosts because cuckoo nestlings evict all host eggs and/or nestlings, thus receiving all of the host parents’ care. Eggs of both warbler species are also targeted by various generalist predators^47–49^. Field work at Delta Marsh was conducted under Canadian Wildlife Service Permit (CWS03 - M013) with animal ethics approval from the University of Manitoba’s Committee on Animal Care (protocol no. F02-008/1, F04-044 ⁄ 45). Field work at the Tomina was conducted under INFS Permit (no. 001658) with animal ethics approval from the University of Manitoba’s Committee on Animal Care (protocol no. F04-044 ⁄ 45). All methods were performed in accordance with the relevant guidelines and regulations.

### Nest defence variables

As part of a larger investigation, we analysed defence responses of yellow and reed warblers recorded to test enemy discrimination abilities^30^ and occurrence of asocial and social learning^32,33^. Defence behaviours were elicited by a series of model presentations during which we placed a model of a perched parasite or predator within 50 cm of a warbler nest before and after a threatening event (i.e. parasitism or egg predation), simulated at the focal nest or at a neighbouring nest. As parasites and nest predators, respectively, cowbird and grackle models were presented to yellow warblers^32^, whereas cuckoo and magpie models to reed warblers^33^. All nests tested were neither parasitized nor depredated when models were presented.

Here, to test effectiveness and selection of nest defence as antiparasite and antipredator responses, we identified relevant behavioural responses elicited by presentations of a series of threatening species models conducted on our focal populations^27,28,32,33^. Specifically, we tested for antiparasite behaviours those responses that changed significantly after a simulated event of parasitism in yellow^27^ and reed^28^ warblers (Supplementary Table 1, Supplementary Fig. 1). We tested for antipredator behaviours that instead resulted as enemy-specific responses in yellow^32^ and reed^33^ warblers (Supplementary Table 1). Model presentations conducted within the above-cited investigations are part of a long-used field-experimental technique, largely shown to be a reliable test to discriminate abilities and information transfer (Supplementary Methods)^12,16,32,50^.

For each behaviour tested for antiparasite or antiparasite defence (Supplementary Table 1), we quantified defence intensity and plasticity. Intensity was the number of times or time spent performing the focal behaviour during 2-min presentations, whereas plasticity was the change in behaviour intensity recorded after a simulated threatening event at own or a neighbour’s nest during two consecutive days^27,28,33^.

### Statistical analyses

We conducted the statistical analyses using Statistica 10 (STATSOFT Inc.).

#### Survival of warbler nests

We define survival of nests in the face of parasitism or egg predation as the time a nest remained unparasitised or not depredated, respectively, until hatching^51–53^. We first determined to what extent warblers were parasitised or their nests were depredated by quantifying the change in the proportion of nests that escaped parasitism or predation, respectively, within an observation period (see below). We used survival analyses^54^, commonly employed to examine ecological data in the form of “time until an event occurs”^55–57^. The distribution of survival times, that is, the survival function, was estimated using the Kaplan-Meier procedure^58,59^. Nests that remained unparasitised or not depredated were defined as having survived (i.e. censored). The observation period for estimating survival to parasitism spanned from the clutch-initiation day to two and three days after clutch completion for yellow and reed warblers, respectively. These windows of observation were determined using the dates during which parasitism was observed in these^27,28^ and other populations^60–62^. Given that eggs are exposed to predation throughout laying and the incubation period, the procedure to determine nest survival to egg predation was the same as described above but with a longer observation period, from laying to the day before hatching for both species. We excluded nests that were parasitized and then depredated as our goal was to disentangle the effects of nest defence serving its dual function, as antiparasite and antipredator strategy.

#### Effectiveness of nest defence phenotypes

In both warbler populations, to test the effect of nest defence on survival to parasitism and egg predation, we ran a Cox model that is analogous to nonparametric multiple regression analysis used to test whether continuous variables predicted survival time^16,58,63^. This model, which permits testing more than one variable as continuous variables, and thus as survival predictors, computes the maximum likelihood parameter estimates and evaluates the overall goodness-of-fit. Hence, we tested whether intensity and plasticity of defence responses (continuous independent variables) predicted nest survival time (dependent variable), thus whether breeders with specific defence phenotypes (see “Nest defence variables”, Supplementary Table 1) better escaped parasitism or predation. For each defence behaviour, intensity phenotypes ranged from weak to strong defenders according to the defence frequency recorded during first exposure to nest threats, whereas plasticity phenotypes ranged from learner to non-learners (*sensu*^31^), according to change of defence intensity recorded after a threatening event. Significant effects of defence phenotypes on nest survival were visualised by survival curves of three models obtained with population mean, minimum and maximum values of the significant defence predictors.

In yellow warblers, Cox models indicated more than one behavioural predictor significantly affected survival to egg predation and therefore we used a Principal Components Analysis^64^ (PCA) to reveal potential alternative defence expressions. Thus, intensity of behavioural predictors was entered to visualise its distribution in the PCA quadrants to indicate segregation among alternative defence strategies (Supplementary Fig. 1).

#### Selection of nest defence phenotypes

Multilevel selection analyses facilitated an examination of relative fitness measures of warblers in relation to defence intensity and plasticity quantified by defence standardised values^21,65^. To quantify selection forces potentially acting on defence phenotypes that predict nest survival, we conducted a multi-level phenotypic selection analysis^21^. This involved multiple linear regressions on correlated traits where linear (β) and non-linear (γ) partial regression coefficients, or selection gradients, show the mode and strength of selection^18,66^.

We treated intensity and plasticity as independent traits, whereas the number of hatchlings was treated as a continuous variable representing an indirect measure of fitness^22^. Defence intensity and plasticity were scored by using the sum of the defence variables that emerged as significant predictors of nest survival from the survival analyses reported above. For yellow warblers, *chip* calls, perch changes and nest-protection behaviour served as intensity phenotype scores, whereas the plasticity phenotype scores included all of the above responses plus strikes directed toward threats. In reed warblers, perch changes and *rasp* calls served as both intensity and plasticity scores. Defence scores were standardized (i.e. z-scores) in relation to population values with their means and variance equal to zero and one, respectively^42,51^. We also standardised the reproductive values of each breeding pair by quantifying the relative fitness (*w*), thus by quantifying the number of hatchlings divided by the mean number of hatchlings in the population for the years studied^13,65,66^.

Following multi-level phenotypic selection analyses, the slope or selection gradients of univariate regressions between one trait and a fitness measure reveal not only whether that trait is under selection, but also its mode and strength^21^. This is straightforward for univariate models^67^, when one trait is tested at a time. When testing more than one trait at a time and, therefore, when complex interactions between phenotypes occur, correct interpretations of selection gradients are difficult. We therefore used spline graphs to depict fitness surfaces where relative fitness is shown as a function of defence intensity and plasticity^65^. Thus, we investigated fitness surfaces, specifically looking at the highest or lowest fitness peaks and the correspondent intensity/plasticity combination values^65,68^. As our hypothesis predicted strong learners as the most effective defence phenotypes to prevent parasitism and predation, we expected, in addition to high effect sizes of linear selection gradients on both intensity and plasticity, a declining surface where the fitness peak corresponded to the highest values of both nest defence intensity and plasticity. Instead, the opposite scenario emerged, with low selection gradients accompanied by a flat fitness surface (i.e. from strong to weak defenders and from learners to non-learners).

### Data availability statement

Authors declare to make materials, data, code, and associated protocols promptly available to readers without undue qualifications.

## Electronic supplementary material

Supplementary Information
