# Supplementary material for: Evolutionary significance of antiparasite, antipredator and learning phenotypes of avian nest defence
Source: Sci Rep. 2018 Jul 12;8:10569. doi: 10.1038/s41598-018-28275-3 (PMC6043525; doi:10.1038/s41598-018-28275-3)
Supplement: Supplementary file 1 — Supplementary Information [file 41598_2018_28275_MOESM1_ESM.docx]

**Supplementary Information**

Evolutionary significance of antiparasite, antipredator and learning phenotypes of avian nest defence

Daniela Campobello and Spencer G. Sealy

| **Yellow warblers** | | **Reed warblers** | |
| --- | --- | --- | --- |
| **Antiparasite** | **Antipredator** | **Antiparasite** | **Antipredator** |
| *seet* calls | *chip* calls | perch changes | perch changes |
| Attacks | *seet* calls | bill snaps | strikes |
| distraction displays | perch changes | *churr* calls | bill snaps |
| out of sight | attacks | *huit* calls | close flights |
|  | displacement activities | *rasp* calls | *churr* calls |
|  |  |  | *huit* calls |
|  |  |  | songs |
|  |  |  | *rasp* calls |
|  |  |  | watching the model |
|  |  |  | out of sight |

**Supplementary Table 1 Nest defence behaviours identified as predictors of nest survival as an antiparasite or antipredator defence response**. Antiparasite defences differed significantly between the first and successive exposures to nest threats when the ability to refine antiparasite responses was tested^28,29^. Antipredator defence were elicited as enemy-specific nest defence responses^34,35^.

**
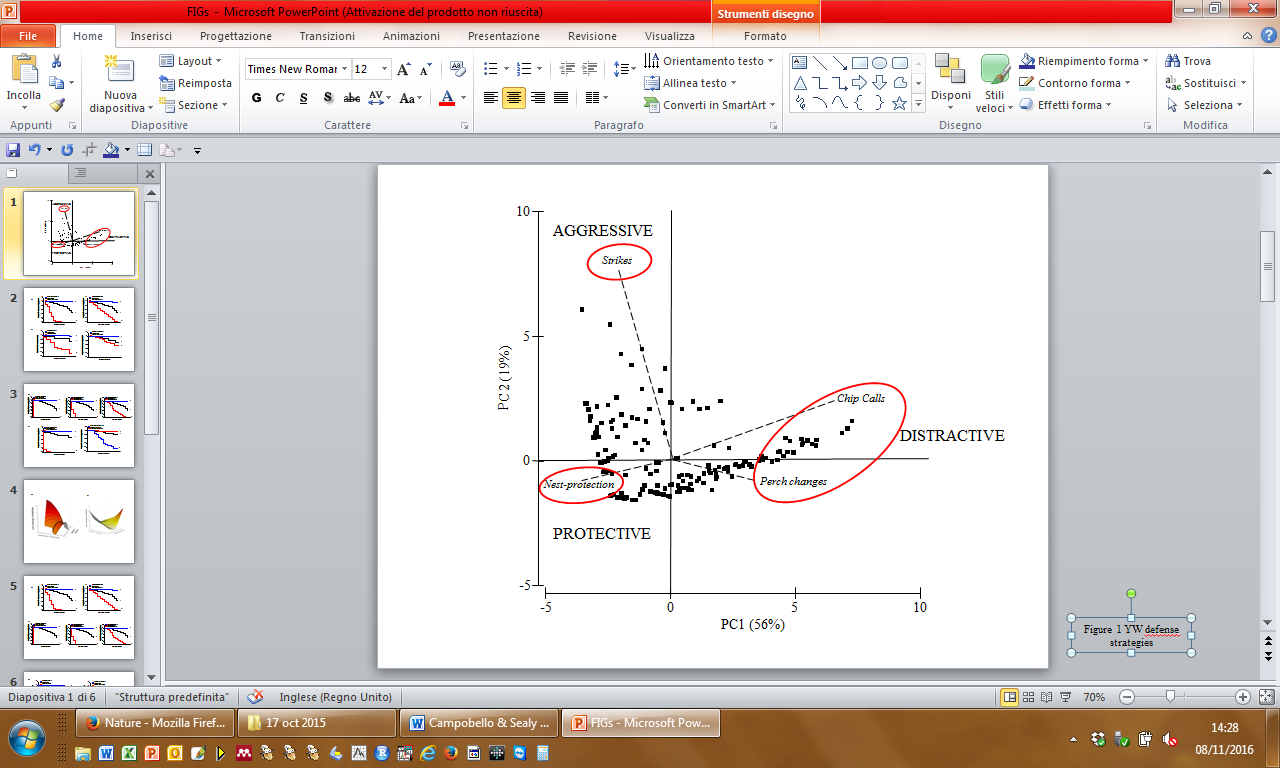
**

**Supplementary Figure 1. Intensity of yellow warbler responses that predict nest survival to egg predation distributed across PCA quadrants**. A PCA explaining 75% of intensity variance (PC1 = 56%, PC2 = 19%) revealed three alternative antipredator strategies, denoted here for brevity, as distractive, protective and aggressive, whose intensity and plasticity correlated with successful avoidance of egg predation.

**Supplementary Methods: Model presentation protocol**

The following descriptions, which summarise the procedure used by Gill and Sealy^34^ and Campobello and Sealy^28,29,35^ are reported here to improve the readability of the present investigation.

Yellow warblers were studied at the Delta Marsh, Manitoba whereas reed warblers at Valli di Mortizzuolo Reserve (hereafter, Tomina (Modena, Italy). Yellow and reed warblers were exposed to models (i.e. taxidermic mounts) with the aim to test their abilities to discriminate among nest predators, brood parasites and nonthreatening species^34,35^, or their individual and social learning abilities^28,29^ measured as behavioural changes that followed a threatening event at their own or a neighbour's nest. Observations were conducted during a 2-min trial from a blind set 5-10 m from the focal nest at least 15 min prior to testing to allow nest owners to habituate to the blind. Models were clipped to the vegetation, facing the nest and approximately 0.5 m from it. After 15 min had elapsed after the first presentation, successive models were similarly presented. We used two models of each type, selecting them via coin toss.

**For the discriminatory tests**^34,35^, yellow warblers were presented with models of a fox sparrow (*Passerella iliaca*), common grackle (*Quiscalus quiscula*) and female cowbird. Fox sparrows are similar in size to cowbirds (32 g vs 39 g, respectively), but differ in plumage^31^. By contrast, common grackles (females, 100 g; males, 127 g) are larger than cowbirds. Reed warblers were presented with models of a cuckoo, rock pigeon (*Columba livia*), and European magpie (*Pica Pica*). Pigeons do not threaten reed warblers or their eggs or young and they are similar to cuckoos in length (31-34 cm versus 32-34 cm, respectively), plumage colouration (medium grey), although pigeons are heavier than cuckoos (200-302 g versus 106-133 g, respectively) ^51^. Magpies threaten nest contents as they feed opportunistically, often depredating passerine young and eggs and occasionally adults ^51^.

**For the learning tests**^34,35^, we simulated parasitism events. Unparasitized and non-depredated nests were presented with models of brood parasites and nonthreatening species at the laying or incubation stage. Trials on yellow warblers were performed between 0500 and 1930 Central Standard Time (CST), and those on reed warblers between 0600 and 1900 Central European Time (CET). Because we predicted a change in the nest defensive responses (see Behavioural Analyses) after a simulated parasitism event, to verify that this change was not due to the experimental protocol *per se* (*i.e.*, sensitization) ^31^, we tested a control group of nests with a model of the brood parasite and with a nonthreatening species for each of two days (Supplementary Tables 2 and 3). Presentations lasted 2 and 5 min on the first and second days, respectively. As the first presentations were reduced to 2 min to avoid problems of habituation with repeated exposures^31^, only the first 2 minutes of the second presentations were analyzed to permit statistical comparisons. Yellow warblers were tested with models of a female cowbird and fox sparrow whereas reed warblers were tested with models of a cuckoo and pigeon. Each nest in the control group was, therefore, exposed on one day to the parasite and control models, and the next day to the same two stimuli (Supplementary Tables 2 and 3).

To assess whether personal experience modified the intensity of the specific responses toward brood parasites, we simulated visits by parasites to host nests using repeated model presentations. Cowbird parasitism and egg removal are usually separate events^11,31^, whereas cuckoos usually remove one host egg seconds before laying their eggs^10^. For this reason, we conducted two separate treatments on yellow warblers and one treatment on reed warblers. Each treatment was performed on different groups of nests, thus each nest was tested with only one treatment. Each treatment consisted of three trial periods: BEFORE, TRAINING, and AFTER (Supplementary Tables 2 and 3). During the BEFORE and AFTER trials, we presented the cowbird or cuckoo model following the same protocol used in the control group, thus with the parasite perched near the nest. The goal of the TRAINING period was to expose hosts to repeated experiences, specific for each treatment. To minimize habituation^30,33^, we performed only two days of training and reduced the duration of each trial to 1 minute. One group of yellow warbler nests was exposed to an experimental parasitism experience, i.e., a cowbird placed on the nest in laying position (Supplementary Table 2). Another group of yellow warbler nests was presented with an experimental egg removal experience where a cowbird model was placed on the nest rim with a warbler egg model affixed on the tip of its bill (Supplementary Table 2). The reed warblers were provided with an experimental parasitism where the cuckoo model, in laying position, was placed on the nest with a warbler egg model affixed on the tip of its bill (Supplementary Table 2).

To determine whether observing conspecifics responding to a brood parasite at their nest triggers a different defensive behaviour, we also simulated parasite visits to host nests using repeated model presentations. The social interaction treatment involved three trial periods: BEFORE, TRAINING, and AFTER. During the BEFORE and AFTER trials,we presented the parasite model following the same protocol used in the control group (Supplementary Tables 2 and 3). The goal of the TRAINING period was to allow yellow warblers and reed warblers to observe conspecifics responding to a parasite at their nest. To provide this experience, 20 minutes before presentation, we placed an experimental nest in a bush or among reeds about 3-5 m from the focal nest. The experimental nest was chosen randomly, via coin toss or draw of sticks of different lengths, among 2-3 natural warbler nests found abandoned during the same nesting season as the experimental trials. For yellow warblers, we used a Sony TMC 5000 EV tape recorder/player and, for reed warblers, a Sony MZ-N710 Mini Disk (MD) recorder/player for the following steps. Concealed in the vegetation, we placed Koss SA/35 loudspeakers connected to the Sony player about 0.5 m from the experimental nest. The blind from which we recorded the warbler response was placed about 2-5 m from the focal nest and 3-5 m from the experimental nest, so that we could see both nests. The pre-presentation set up lasted around 3 min from the time we arrived in the nest area. Once at least 20 min elapsed, we placed one parasite and two warbler models about 0.5 m from the experimental nest and I broadcasted *seet* and *rasp* calls to yellow and reed warblers, respectively, during the 2-min trial. We chose these vocalizations because they were preferentially uttered toward cowbirds^25^ and cuckoos^28^, respectively. The perched parasite was mounted in the same fashion as in the control presentations. The two warblers were mounted in aggressive posture with wings and bills open, feathers on the crown ruffed, and one model was placed on the back of the parasite model to simulate natural attacks as photographed in former investigations^37^; Supplementary Tables 2 and 3). Playbacks of alarm calls were recorded at the beginning of nesting seasons during presentations of parasite models at nests not included in the subsequent experiments. Vocalizations were recorded with the Sony recorder and Sennheiser K3-U directional microphone placed about 1 m from the focal nest. We chose s*eet* and *rasp* call recordings from among those with minimal background noise and reverberation to make two 5-min tracks. The tracks were transferred to 90-min TDK Type I audio cassettes and Sony premium 80 MDs using their respective Sony recorder/players and played back during the 2-min training trials. S*eet* and *rasp* calls were broadcast at amplitudes of 70 dB and 60 dB SPL (Sound Pressure Level), respectively, at 3-5 m from the focal nest, calibrated from about 10 calls/nest measured at 2-5 m from the warblers by a Realistic 33-2050 sound level meter (weighting C, response low; n = number of nests, *seet* calls: mean ± sd = 68 ± 5 dB, n = 5 ; *rasp* calls: 59 ± 6 dB, n = 4) ^25^.

The training trials were conducted over two consecutive days, therefore, each nest was exposed the first day to the BEFORE, the second and the third days to the TRAINING, and on the fourth day to the AFTER trials. Also the control groups received four model presentations although with no training (Supplementary Tables 2 and 3).

**Supplementary Table 2**. Experimental and control treatments to which four groups of yellow warblers were exposed at Delta Marsh (MB, Canada).

| Day | Trial | Parasitism | Egg Removal | Social Interaction | Control |
| --- | --- | --- | --- | --- | --- |
| 1 | BEFORE | Perched cowbird ^A^ | Perched cowbird ^A^ | Perched cowbird ^A^ | Perched cowbird ^A^ |
|  |  |  |  |  | Perched sparrow ^B^ |
| 2-3 | TRAINING | Parasitizing cowbird ^C^ | Egg removing cowbird ^D^ | Warblers defending from cowbird ^E^ + *seet* call playback | No training |
| 4 | AFTER | Perched cowbird ^A^ | Perched cowbird ^A^ | Perched cowbird ^A^ | Perched cowbird ^A^ |
|  |  |  |  |  | Perched sparrow ^B^ |


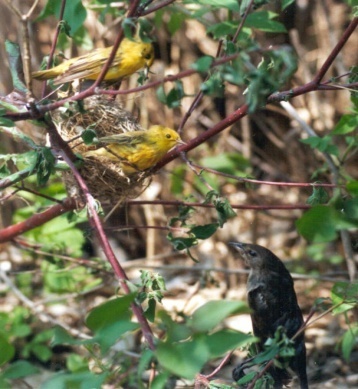

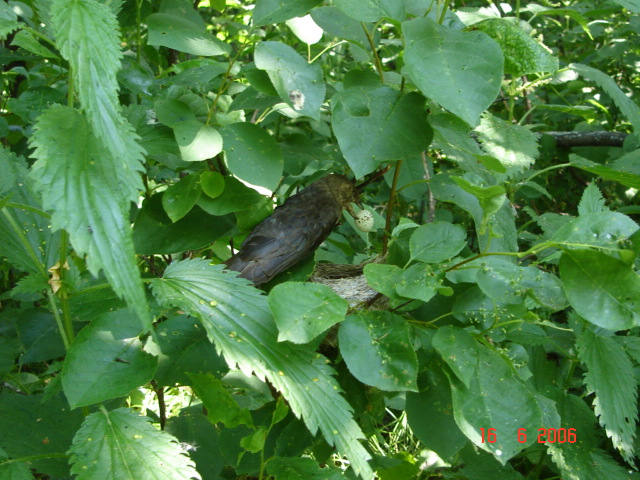

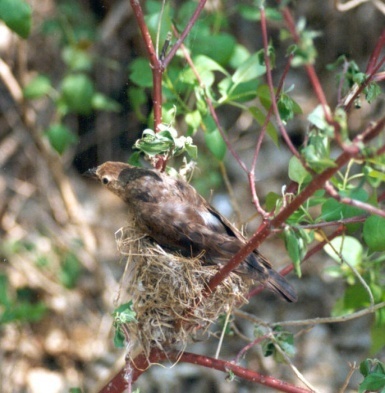

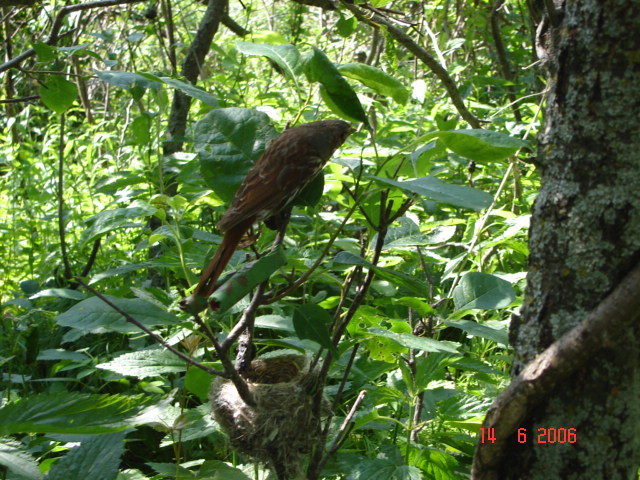

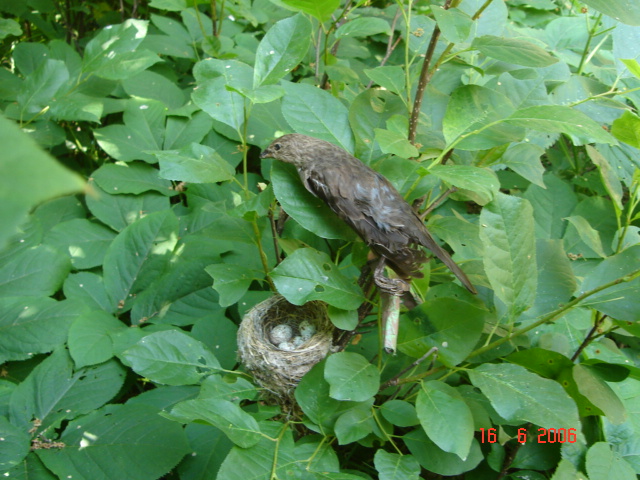


E

D

C

B

A

**Supplementary Table 3**. Experimental and control treatments to which three groups of Reed Warblers were exposed at the Tomina, (Italy).

| Day | Trial | Parasitism | Social Interaction | Control |
| --- | --- | --- | --- | --- |
| 1 | BEFORE | Perched cuckoo ^A^ | Perched cuckoo ^A^ | Perched cuckoo ^A^ |
|  |  |  |  | Perched pigeon ^B^ |
| 2-3 | TRAINING | Parasitizing cuckoo ^C^ | Warblers defending from cuckoo ^D^ + *rasp* call playback | No training |
| 4 | AFTER | Perched cuckoo ^A^ | Perched cuckoo ^A^ | Perched cuckoo ^A^ |
|  |  |  |  | Perched pigeon ^B^ |


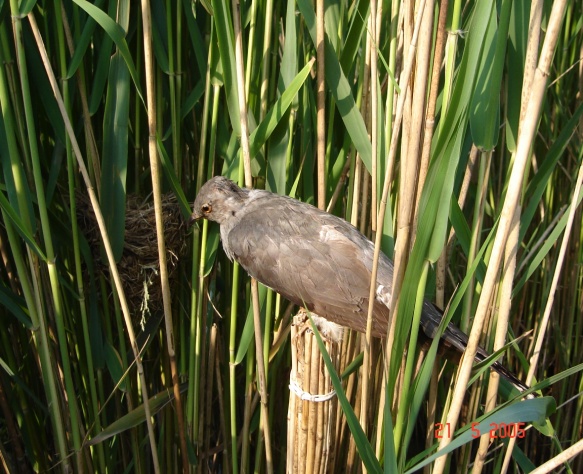

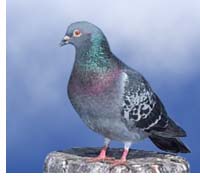

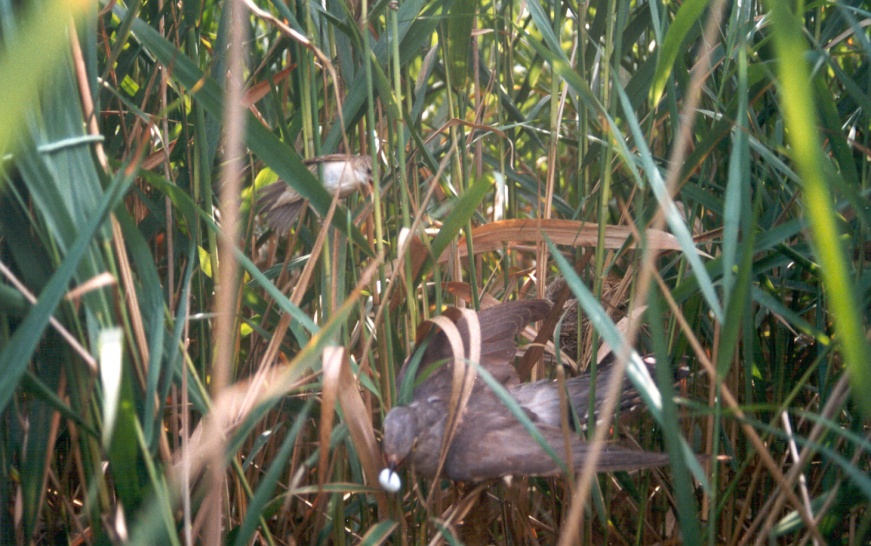

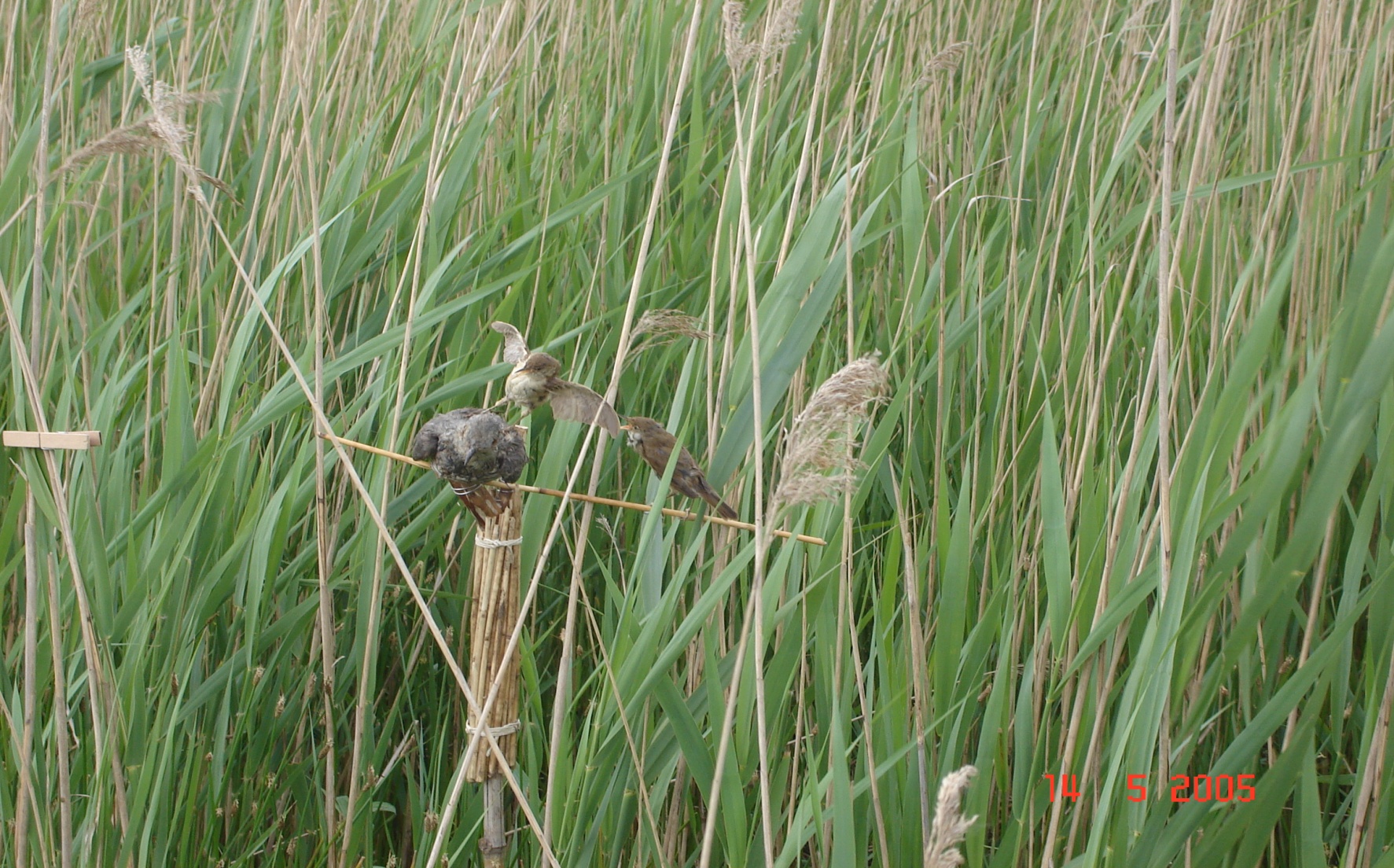


B

C

D

A
